# Supplementary material for: Psychometric properties of the item-reduced version of the comprehensive general parenting questionnaire for caregivers of preschoolers in a Finnish context
Source: PLoS One. 2022 Aug 4;17(8):e0270869. doi: 10.1371/journal.pone.0270869 (PMC9352000; doi:10.1371/journal.pone.0270869)
Supplement: S1 Table — (DOCX) [file pone.0270869.s001.docx]

S1 Table. Result from the statistical analyses for the reduction process, and reasons others than statistical for excluding/including items in the reduced 29-items version

| **Construct and item** | **Dimension^a^** | **Mean****^b^** | **SD** | **29-items version** | **22-items version** | **Corrected Item-Total Correlation (separated by construct)** | **Cronbach's alpha if Item Deleted (separated by construct)** | **Skewness** | **IRT^c^** | **CFA estimate^d^** | **Other than statistical reasons for exclusion or inclusion of an item** |
| --- | --- | --- | --- | --- | --- | --- | --- | --- | --- | --- | --- |
| ***Construct: Behavioral control*** |  |  |  |  |  |  |  |  |  |  |  |
| 13. I expect my child to follow our family rules | EB | 4,38 | 0,58 | yes | yes | 0,40 | 0,72 | -0,50 | 1,01 | 0,28 |  |
| 18. I require my child to behave in certain ways | EB | 3,39 | 0,88 |  |  | 0,21 | 0,74 | -0,69 | 0,03 | 0,22 |  |
| 27. I correct my child when he/she breaks the rules | NP | 4,44 | 0,53 |  |  | 0,39 | 0,72 | -0,11 | 1,39 | 0,23 |  |
| 31. When I correct my child’s behavior, I explain why | NP | 4,41 | 0,61 |  |  | 0,17 | 0,74 | -0,82 | 1,22 | 0,24 |  |
| 33. When my child and I are at home together, I frequently check on what he/she is doing | M | 3,10 | 1,14 |  |  | 0,36 | 0,72 | -0,06 | 0,18 | 0,49 |  |
| 38. I pay close attention to where my child is | M | 3,64 | 0,99 | yes | yes | 0,40 | 0,72 | -0,55 | 0,29 | 0,50 |  |
| 48. When my child misbehaves, I point out what he/she did wrong | NP | 4,45 | 0,60 |  |  | 0,30 | 0,73 | -0,91 | 0,99 | 0,22 |  |
| **Construct and item** | **Dimension^a^** | **Mean^b^** | **SD** | **29-items version** | **22-items version** | **Corrected Item-Total Correlation (separated by construct)** | **Cronbach's alpha if Item Deleted (separated by construct)** | **Skewness** | **IRT^c^** | **CFA estimate^d^** | **Other than statistical reasons for exclusion or inclusion of an item** |
| 52. I make sure I know where my child is at all times | M | 3,99 | 1,01 | yes | yes | 0,50 | 0,70 | -1,17 | 0,46 | 0,60 |  |
| 54. I watch my child to make sure he/she behaves appropriately | M | 2,96 | 1,06 |  |  | 0,40 | 0,72 | -0,10 | 0,20 | 0,41 |  |
| 55. I am aware of what my child is doing when he/she is at home | M | 3,99 | 0,87 |  |  | 0,41 | 0,71 | -0,89 | 0,51 | 0,44 | We concluded, 2 items for "Monitoring" is enough. We included 38 and by discussions 52 was included and 55 was left out. |
| 56. I have clear expectations for how my child should behave | EB | 3,70 | 0,80 |  |  | 0,47 | 0,71 | -0,91 | 0,42 | 0,38 | We concluded that 3 items describing expected behaviour is enough, and decided that 68 describes the expectations better than 56 which was left out. |
| 62. I teach my child to follow rules | EB | 4,43 | 0,67 | yes | yes | 0,36 | 0,72 | -1,22 | 1,34 | 0,32 |  |
| 68. I make sure that my child understands what I expect of him/her | EB | 3,86 | 0,74 | yes |  | 0,37 | 0,72 | -0,56 | 0,78 | 0,32 |  |
| ***Construct: Coercive control*** |  |  |  |  |  |  |  |  |  |  |  |
| 4. When my child does something that is not allowed, I do not talk to him/her for a while | PC | 1,43 | 0,82 | yes |  | 0,30 | 0,62 | 2,36 | -0,16 | 0,21 | Two items needed to reflect psychological control. An agreement of items 4 & 30, the content in them were perceived to be understood by a child. |
| **Construct and item** | **Dimension^a^** | **Mean^b^** | **SD** | **29-items version** | **22-items version** | **Corrected Item-Total Correlation (separated by construct)** | **Cronbach's alpha if Item Deleted (separated by construct)** | **Skewness** | **IRT^c^** | **CFA estimate^d^** | **Other than statistical reasons for exclusion or inclusion of an item** |
| 10. I want my child to always obey me | AC | 3,55 | 0,96 | yes | yes | 0,44 | 0,60 | -0,74 | 0,24 | 0,58 |  |
| 14. I tell my child I am very disappointed, when he/she does not act appropriately | PC | 3,35 | 1,11 |  |  | 0,36 | 0,62 | -0,25 | 0,13 | 0,56 |  |
| 19. I spank my child when he/she does something wrong | PP | 1,04 | 0,23 |  |  | 0,24 | 0,64 | 6,15 | -0,02 | 0,02 |  |
| 23. I place a lot of emphasis on obedience in my child | AC | 3,44 | 0,89 | yes | yes | 0,50 | 0,59 | -0,54 | 0,11 | 0,60 |  |
| 30. I make my child feel bad when he/she does not meet my expectations | PC | 1,81 | 0,90 | yes |  | 0,28 | 0,63 | 1,17 | -0,21 | 0,23 | Two items needed to reflect psychological control. An agreement of items 4 & 30, the content in them were perceived to be understood by a child. |
| 34. I spank my child when he/she is disobedient | PP | 1,02 | 0,19 |  |  | 0,22 | 0,64 | 8,86 | -0,09 | 0,02 |  |
| 37. I make sure my child knows everything I do for him/her | PC | 2,40 | 0,93 |  |  | 0,32 | 0,62 | 0,26 | 0,12 | 0,46 |  |
| 43. I use physical punishment to discipline my child | PP | 1,20 | 0,53 |  |  | 0,12 | 0,65 | 3,06 | -0,11 | 0,03 |  |
|  |  |  |  |  |  |  |  |  |  |  |  |
| **Construct and item** | **Dimension^a^** | **Mean^b^** | **SD** | **29-items version** | **22-items version** | **Corrected Item-Total Correlation (separated by construct)** | **Cronbach's alpha if Item Deleted (separated by construct)** | **Skewness** | **IRT^c^** | **CFA estimate^d^** | **Other than statistical reasons for exclusion or inclusion of an item** |
| 60. I let my child know that I am the boss in our house | AC | 3,51 | 1,08 |  |  | 0,28 | 0,63 | -0,56 | 0,22 | 0,40 |  |
| 61. I spank my child when he/she is behaving inappropriately | PP | 1,02 | 0,19 |  |  | 0,20 | 0,64 | 8,86 | 0,07 | 0,02 |  |
| 64. I do not allow my child to get angry with me | AC | 1,43 | 0,64 |  |  | 0,24 | 0,63 | 1,49 | -0,26 | 0,18 |  |
| ***Construct: Nurturance*** |  |  |  |  |  |  |  |  |  |  |  |
| 1. I encourage my child to be curious and to explore things | AS | 4,47 | 0,58 |  |  | 0,26 | 0,75 | -0,73 | 0,34 | 0,10 |  |
| 5. I find time to play with my child | I | 3,92 | 0,72 |  |  | 0,18 | 0,76 | -1,10 | 0,16 | 0,12 |  |
| 7. I know exactly when things are not going very well for my child | R | 4,38 | 0,72 | yes | yes | 0,34 | 0,74 | -1,75 | 0,71 | 0,23 |  |
| 9. I praise my child when he/she does something good | SR | 4,82 | 0,43 |  |  | 0,37 | 0,74 | -2,71 | 1,42 | 0,18 |  |
| 17. I let my child make his/her own choices as long as they are safe | AS | 4,28 | 0,52 | yes | yes | 0,36 | 0,74 | -0,04 | 0,95 | 0,22 |  |
| 21. I say something nice to my child as a reward for good behavior | SR | 4,42 | 0,72 | yes | yes | 0,26 | 0,75 | -1,40 | 0,67 | 0,21 |  |
| **Construct and item** | **Dimension^a^** | **Mean^b^** | **SD** | **29-items version** | **22-items version** | **Corrected Item-Total Correlation (separated by construct)** | **Cronbach's alpha if Item Deleted (separated by construct)** | **Skewness** | **IRT^c^** | **CFA estimate^d^** | **Other than statistical reasons for exclusion or inclusion of an item** |
| 26. When my child does his/her best, I praise him/her | SR | 4,82 | 0,40 | yes |  | 0,42 | 0,74 | -1,96 | 2,46 | 0,18 | Higher discrimination (IRT) and we needed another item describing ST |
| 32. I feel good about the relationship I have with my child | R | 4,61 | 0,58 |  |  | 0,47 | 0,73 | -1,38 | 0,96 | 0,27 | Two items enough to describe R, the item was perceived "odd" in relation to items 7 and 67. |
| 36. I tell my child how much I appreciate it when he/she helps me | SR | 4,72 | 0,51 |  |  | 0,37 | 0,74 | -1,87 | 1,38 | 0,22 |  |
| 40. I spend a lot of time with my child | I | 4,28 | 0,73 | yes | yes | 0,41 | 0,74 | -1,22 | 0,70 | 0,40 |  |
| 44. I easily find a way to make time for my child | I | 3,85 | 0,83 | yes | yes | 0,34 | 0,74 | -0,58 | 0,34 | 0,33 |  |
| 49. My child and I have warm affectionate moments together | R | 4,84 | 0,40 |  |  | 0,39 | 0,74 | -2,39 | 1,26 | 0,12 |  |
| 51. I encourage my child to try things on his/her own before I help | AS | 4,57 | 0,61 |  |  | 0,30 | 0,75 | -1,87 | 0,52 | 0,09 |  |
| 59. I encourage my child to approach things his/her own way, even if it means more work for me | AS | 4,16 | 0,72 | yes | yes | 0,43 | 0,73 | -0,63 | 0,90 | 0,36 |  |
| 66. I praise my child when he/she deserves it | SR | 4,84 | 0,37 |  |  | 0,32 | 0,75 | -1,85 | 1,36 | 0,12 |  |
| **Construct and item** | **Dimension^a^** | **Mean^b^** | **SD** | **29-items version** | **22-items version** | **Corrected Item-Total Correlation (separated by construct)** | **Cronbach's alpha if Item Deleted (separated by construct)** | **Skewness** | **IRT^c^** | **CFA estimate^d^** | **Other than statistical reasons for exclusion or inclusion of an item** |
| 69. I find it interesting and educational to be with my child for long periods | I | 4,43 | 0,73 |  |  | 0,32 | 0,74 | -1,15 | 0,49 | 0,30 |  |
| ***Construct: Overprotection*** |  |  |  |  |  |  |  |  |  |  |  |
| 12. Every free minute I have, I spend with my child | EI | 3,38 | 1,09 | yes | yes | 0,32 | 0,55 | -0,30 | 0,23 | 0,56 |  |
| 16. I always help my child with everything he/she does | EI | 2,97 | 1,05 | yes | yes | 0,40 | 0,52 | 0,09 | 0,29 | 0,68 |  |
| 41. When my child cannot find something, I stop what I am doing to find it before he/she gets too upset | EI | 2,94 | 1,09 | yes | yes | 0,40 | 0,52 | 0,06 | 0,16 | 0,50 | We decided to exclude only those with poor results in the statistical analyses (Skewness, IRT and ACF) as we had a special interest in this construct |
| 45. I do not let my child stay with our family or friends without me or my spouse present | EI | 1,34 | 0,69 |  |  | 0,16 | 0,60 | 2,84 | -0,06 | 0,11 |  |
| 47. I do not let my child get involved in activities or tasks where he/she might get hurt | EI | 2,13 | 1,05 | yes | yes | 0,32 | 0,55 | 0,80 | 0,03 | 0,30 |  |
| 50. I always choose what toy my child should play with | EI | 1,13 | 0,38 |  |  | 0,24 | 0,59 | 3,15 | -0,3 | 0,07 |  |
| **Construct and item** | **Dimension^a^** | **Mean^b^** | **SD** | **29-items version** | **22-items version** | **Corrected Item-Total Correlation (separated by construct)** | **Cronbach's alpha if Item Deleted (separated by construct)** | **Skewness** | **IRT^c^** | **CFA estimate^d^** |  |
| ***Construct: Structure*** |  |  |  |  |  |  |  |  |  |  |  |
| 2. When I tell my child I will do something, I do it | C | 4,29 | 0,66 | yes |  | 0,38 | 0,73 | -1,25 | 0,80 | 0,31 |  |
| 3. I make sure my child has enough time to get ready for activities | O | 3,86 | 0,88 |  |  | 0,30 | 0,73 | -1,11 | 0,34 | 0,34 | After discussions we excluded item 3 and kept item 35, which was perceived as better describing “Structure” |
| 8. I use clear and consistent messages when I tell my child to do something | C | 4,31 | 0,55 | yes |  | 0,46 | 0,73 | -0,19 | 1,06 | 0,29 |  |
| 11. I try not to change the rules at home very often | C | 4,43 | 0,66 |  |  | 0,40 | 0,73 | -1,34 | 0,80 | 0,34 | Dropped after discussions with colleagues which perceived this item odd |
| 15. When my child is struggling with something, I try to find ways to help him/her | S | 4,60 | 0,54 |  |  | 0,23 | 0,74 | -0,84 | 1,21 | 0,16 |  |
| 20. I have a hard time consistently enforcing rules with my child | ID | 4,08 | 0,94 |  |  | 0,42 | 0,72 | 1,13 | 0,28 | 0,40 |  |
| 22. I try not to forget the promises I make to my child | C | 4,64 | 0,52 |  |  | 0,33 | 0,73 | -0,95 | 1,32 | 0,22 |  |
| 25. I do not always follow through when I threaten to discipline my child | ID | 2,95 | 1,14 |  |  | 0,40 | 0,73 | -0,27 | 0,20 | 0,52 |  |
| 28. I try to make sure that my child has a regular schedule from day to day | O | 4,19 | 0,87 | yes | yes | 0,31 | 0,73 | -1,14 | 0,44 | 0,38 |  |
| **Construct and item** | **Dimension^a^** | **Mean^b^** | **SD** | **29-items version** | **22-items version** | **Corrected Item-Total Correlation (separated by construct)** | **Cronbach's alpha if Item Deleted (separated by construct)** | **Skewness** | **IRT^c^** | **CFA estimate^d^** | **Other than statistical reasons for exclusion or inclusion of an item** |
| 35. I organize my child’s week so that it follows a regular, predictable pattern | O | 3,90 | 1,02 | yes |  | 0,38 | 0,73 | -0,94 | 0,41 | 0,51 |  |
| 39. I threaten discipline more often than I actually give it | ID | 3,35 | 1,25 |  |  | 0,37 | 0,73 | 0,23 | 0,15 | 0,43 |  |
| 42. I encourage my child to pick up his/her toys | O | 4,50 | 0,65 |  |  | 0,25 | 0,74 | -1,32 | 0,51 | 0,16 |  |
| 57. When my child has difficulties, I help him/her | S | 4,55 | 0,52 | yes | yes | 0,28 | 0,74 | -0,45 | 1,96 | 0,23 |  |
| 58. I make sure my child is at activities on time | O | 4,57 | 0,59 |  |  | 0,30 | 0,74 | -1,36 | 0,66 | 0,20 |  |
| 63. When my child has a problem, I help him/her figure out what to do about it | S | 4,50 | 0,55 | yes | yes | 0,29 | 0,74 | -0,45 | 1,41 | 0,23 |  |
| 65. There are times I just do not have energy to make my child behave as he/she should | ID | 3,02 | 1,30 |  |  | 0,34 | 0,74 | 0,02 | 0,11 | 0,44 |  |

^a^ Dimensions within the five constructs: EB Expectations for behavior, NP Non-physical punishment, M Monitoring, PC Psychological control, PP Physical punishment, AC Authoritarian control, AS Autonomy support, I Involvement, R Responsiveness, SR Social rewarding, AS Autonomy support, EI Excessive involvement, C Consistency, O Overprotection, S Scaffolding, ID Inconsistent discipline.

^b^ Coding range 1–5

^c^ IRT Item Response Theory (the discrimination parameter)

^d^ CFA Confirmatory Factor Analyses
