# Supplementary material for: Psychometric properties of the item-reduced version of the comprehensive general parenting questionnaire for caregivers of preschoolers in a Finnish context
Source: PLoS One. 2022 Aug 4;17(8):e0270869. doi: 10.1371/journal.pone.0270869 (PMC9352000; doi:10.1371/journal.pone.0270869)
Supplement: Attachment — Submitted filename: Response to reviewers.docx [file pone.0270869.s002.docx]

PONE-D-21-32971

Psychometric properties of the item-reduced version of the Comprehensive General Parenting Questionnaire for caregivers of preschoolers in a Finnish context

PLOS ONE

Dear Dr. Ray,

Thank you for submitting your manuscript to PLOS ONE. After careful consideration, we feel that it has merit but does not fully meet PLOS ONE’s publication criteria as it currently stands. Therefore, we invite you to submit a revised version of the manuscript that addresses the points raised during the review process.

Apologies for the long time spent processing this first version of your paper. Unfortunately, it was really difficult to locate suitable (and available) reviewers for this paper. Anyway, I got a first review report, with which (given the situation) I will go straightforward to you, in order to ask you for some (minor) revisions, in order to reconsider the acceptability of the paper.

Although most of these changes are rather miscellaneous, please make sure to address all the comments provided by the referee, responding to them in the rebuttal letter.

Please submit your revised manuscript by Apr 21 2022 11:59PM. If you will need more time than this to complete your revisions, please reply to this message or contact the journal office at [plosone@plos.org](mailto:plosone@plos.org). When you're ready to submit your revision, log on to https://www.editorialmanager.com/pone/ and select the 'Submissions Needing Revision' folder to locate your manuscript file.

Please include the following items when submitting your revised manuscript:

- A rebuttal letter that responds to each point raised by the academic editor and reviewer(s). You should upload this letter as a separate file labeled 'Response to Reviewers'.
- A marked-up copy of your manuscript that highlights changes made to the original version. You should upload this as a separate file labeled 'Revised Manuscript with Track Changes'.
- An unmarked version of your revised paper without tracked changes. You should upload this as a separate file labeled 'Manuscript'.

If you would like to make changes to your financial disclosure, please include your updated statement in your cover letter. Guidelines for resubmitting your figure files are available below the reviewer comments at the end of this letter.

If applicable, we recommend that you deposit your laboratory protocols in protocols.io to enhance the reproducibility of your results. Protocols.io assigns your protocol its own identifier (DOI) so that it can be cited independently in the future. For instructions see: <https://journals.plos.org/plosone/s/submission-guidelines#loc-laboratory-protocols>. Additionally, PLOS ONE offers an option for publishing peer-reviewed Lab Protocol articles, which describe protocols hosted on protocols.io. Read more information on sharing protocols at <https://plos.org/protocols?utm_medium=editorial-email&utm_source=authorletters&utm_campaign=protocols>.

We look forward to receiving your revised manuscript.

Kind regards,

Sergio A. Useche, Ph.D.
Academic Editor
PLOS ONE

Journal Requirements:

When submitting your revision, we need you to address these additional requirements.

1. Please ensure that your manuscript meets PLOS ONE's style requirements, including those for file naming. The PLOS ONE style templates can be found at
   <https://journals.plos.org/plosone/s/file?id=wjVg/PLOSOne_formatting_sample_main_body.pdf> and 
   <https://journals.plos.org/plosone/s/file?id=ba62/PLOSOne_formatting_sample_title_authors_affiliations.pdf>

We have named the files as instructed

2. Please provide additional details regarding participant consent. In the ethics statement in the Methods and online submission information, please ensure that you have specified what type you obtained (for instance, written or verbal, and if verbal, how it was documented and witnessed). If your study included minors, state whether you obtained consent from parents or guardians. If the need for consent was waived by the ethics committee, please include this information.

We have clarified the details regarding participation in studies 1 and 2 in the manuscript (lines 128-136). In study 1, the respondents were informed as written text in the beginning of the online questionnaire that by answering they were agreeing to participate in the study. We present the information about the written consent in study 2, as well. We have also corrected the details in the online submission.

3. Thank you for stating the following in the Acknowledgments Section of your manuscript:

The DAGIS project and this study was financially supported by Folkhälsan Research Center, University of Helsinki, The Ministry of Education and Culture in Finland, The Ministry of Social Affairs and Health, The Academy of Finland (Grant: 285439, 315816), the Päivikki and Sakari Sohlberg foundation, and the Medicinska Föreningen Liv och Hälsa.

Please note that funding information should not appear in the Acknowledgments section or other areas of your manuscript. We will only publish funding information present in the Funding Statement section of the online submission form. 
Please remove any funding-related text from the manuscript and let us know how you would like to update your Funding Statement. Currently, your Funding Statement reads as follows:

The DAGIS project and this study was financially supported by Folkhälsan Research Center, University of Helsinki, The Ministry of Education and Culture in Finland (ER), The Ministry of Social Affairs and Health (ER), The Academy of Finland (Grant: 285439, 315816) (ER), the Päivikki and Sakari Sohlberg foundation (ER), and the Medicinska Föreningen Liv och Hälsa (ER). The funders had no role in study design, data collection and analysis, decision to publish, or preparation of the manuscript.

Please include your amended statements within your cover letter; we will change the online submission form on your behalf.

Thank you for this comment. We have excluded this information from the manuscript. In the cover letter we have included what should be stated about the funding.

4. We note that you have stated that you will provide repository information for your data at acceptance. Should your manuscript be accepted for publication, we will hold it until you provide the relevant accession numbers or DOIs necessary to access your data. If you wish to make changes to your Data Availability statement, please describe these changes in your cover letter and we will update your Data Availability statement to reflect the information you provide.

We have described how data is available in the cover letter.

5. Please include captions for your Supporting Information files at the end of your manuscript, and update any in-text citations to match accordingly. Please see our Supporting Information guidelines for more information: http://journals.plos.org/plosone/s/supporting-information.

We have included captions about our Supplementary Table at the end of the manuscript. In-text citations are also updated.

6. Please review your reference list to ensure that it is complete and correct. If you have cited papers that have been retracted, please include the rationale for doing so in the manuscript text, or remove these references and replace them with relevant current references. Any changes to the reference list should be mentioned in the rebuttal letter that accompanies your revised manuscript. If you need to cite a retracted article, indicate the article’s retracted status in the References list and also include a citation and full reference for the retraction notice.
[Note: HTML markup is below. Please do not edit.]

We are sorry, but we do not understand the comments about citing retracted papers?

We have added references in the manuscript. These references are mentioned in the rebuttal letter when discussing the comments with the reviewer.

Reviewers' comments:

Reviewer's Responses to Questions

**Comments to the Author**

Reviewer #1: This study assesses the factorial structure of the reduced item version of the Comprehensive General Parenting Questionnaire (CGPQ), an instrument that evaluates five general parenting constructs. The manuscript presents important practical implications, due to the need of assessing the validity of this type of questionnaires, considering the facility of their administration in comparison with more extended versions.
The introduction adequately presents the previous studies performed through different questionnaires framed within this field of study. 1)However, I believe that the references are scarce, as the issue needs to be contextualized and the study requires an adequate theoretical framework. Therefore, I suggest a further effort to complement this section.

Answer: Thank you for the comment. We have complemented several sections in the introduction to conceptualize the issue and the larger theoretical framework. We added references related to these issues throughout the introduction. Please see the changes in lines 62-68, 79-82, 87-88, and 92-95.

The added references in the introduction are:

Howe AS, Heath A-LM, Lawrence J, Galland BC, Gray AR, Taylor BJ, et al. (2017). Parenting style and family type, but not child temperament, are associated with television viewing time in children at two years of age. PLoS ONE 12(12): e0188558. https://doi.org/10.1371/journal.pone.0188558

Van der Geest KE, Mérelle SYM, Rodenburg G, Van de Mheen, Renderset CM. Cross-sectional associations between maternal parenting styles, physical activity and screen sedentary time in children. BMC Public Health (2017) 17:753 DOI 10.1186/s12889-017-4784-8

Burnett AJ, Lamb KE, Spence AC, Lacy KE, Worsley A. Parenting style as a predictor of dietary score change in children from 4 to 14 years of age. Findings from the Longitudinal Study of Australian Children. Public Health Nutr. 2021 Dec;24(18):6058-6066. doi: 10.1017/S1368980021003062. Epub 2021 Jul 23. PMID: 34296665.

Philips N, Sioen I, Michels N, Sleddens E, De Henauw S. The influence of parenting style on health related behavior of children: findings from the ChiBS study. International Journal of Behavioral Nutrition and Physical Activity 2014, 11:95 http://www.ijbnpa.org/content/11/1/95

Gerards SMPL, Niermann C, Gevers DWM, Eussen N, Kremers SPJ. Context matters! The relationship between mother-reported family nutrition climate, general parenting, food parenting practices and children’s BMI. BMC Public Health (2016) 16:1018 DOI 10.1186/s12889-016-3683-8.

The methodology is adequately presented, but I suggest 2) including some table or figure clarifying the dimensions that should be obtained from the questionnaire.

Answer: In the S1 Table (Supplementary Information) we present in the first column each of the five construct and their included items. The second column includes all the dimensions. We prefer to have this table as a Supplementary file, as it is very large, including the psychometrics of each item in the 69-item scale. We have in the results section added a sentence about the S1 Table (lines 280-283), and in the end of the manuscript we added a notice about that the manuscript includes Supplementary Information. In addition, the dimensions are presented in both Table 5 and Figure 1 in the main manuscript.

Likewise, it would also be convenient to 3) explain the psychometric properties of each item through a table, as well as including a figure explaining the correspondence between the items and the dimensions of the questionnaire (the following article may be useful for this purpose: <https://doi.org/10.1016/j.trf.2018.08.003>).

Answer: Thank you for your suggestion. In our study the psychometric properties of the scale were examined by the item response theory, the AFC to evaluate the factor structure, internal consistency, and internal validity as described in the text (Added in the beginning of the discussion in lines 324-325.  And as suggested, we present in line 340 a figure named Figure 1. Standard estimation of Confirmatory Factor Analysis (CFA) for the 22 items (Study 2). All estimates p<0.001. The figure is pointing out the correspondence between the items and each construct of the questionnaire.

4) Moreover, the average variance extracted (AVE) and the adjustment indexes of the scale must be included.

Answer: Thank you for this important suggestion.

We have added the AVE and following changes have been done in the manuscript.

- We have in the statistical part presented that we will examine the AVE and we have added a reference as well, lines 217-219: “We evaluated the convergent validity by the average variance extracted (AVE) for each latent factor. AVE over 0.50 is recommended for each construct, indicating a good convergent validity [Hair J 2010].”
- Added reference: Hair J., Black W., Babin B., Anderson R. (2010). *Multivariate Data Analysis*, 7th Edn Upper Saddle River, NJ: Prentice-Hall, Inc.

In addition, we calculated and included the AVE in the result section (Lines 293-297 and Table 5).

- The adjustment indexes were presented in Table 6. "Detailed goodness-of-fit of models for CFA of three different versions evaluated in Study 1 and Study 2 data".
- New text in the document: “The AVE for ACF estimated in Study 1, shown value over 0.5 for all constructs, indicating a good convergent validity, and that no other item needed to be deleted (Table 5). The AVEs for the constructs estimated in Study 2 were even higher, with all values over 0.80, demonstrating excellent results. The standard estimation obtained for Study 2 are presented in Fig 1, in which all estimated value with a significance p<0.001.”

The discussion summarizes the main findings of the study, but 5) this section must also be complemented with other studies that may serve to contrast the results.

Answer: Overall, we have elaborated the discussion. We now refer to other studies, conducted with the CGPQ instrument, but also to other studies which have examined psychometric properties of an instrument.

The added sections about other studies are in lines 346-349, 364-368.

Added references:

Gevers DW, van Assema P, Sleddens EF, de Vries NK, Kremers SP. Associations between general parenting, restrictive snacking rules, and adolescent's snack intake. The roles of fathers and mothers and interparental congruence. Appetite. 2015 Apr;87:184-91. doi: 10.1016/j.appet.2014.12.220. Epub 2014 Dec 30. PMID: 25555538.

Bohman B, Rasmussen F, Ghaderi A. Development and psychometric evaluation of a context-based parental self-efficacy instrument for healthy dietary and physical activity behaviors in preschool children. International Journal of Behavioral Nutrition and Physical Activity (2016) 13:110 DOI 10.1186/s12966-016-0438-y.

6) In addition, the practical implications of the research could be included in the discussion/conclusion.

Answer: Thank you for the comment. We have added a section about practical implications and proposed some further studies. Please see lines 389-401.

For what concerns the style and writing of the manuscript, they are adequate.

Thank you!
